# APRIL and BAFF: novel biomarkers for central nervous system lymphoma

**DOI:** 10.1186/s13045-019-0796-4

**Published:** 2019-10-15

**Authors:** Matthias Mulazzani, Marion Huber, Sabine Borchard, Sigrid Langer, Barbara Angele, Elisabeth Schuh, Edgar Meinl, Martin Dreyling, Tobias Birnbaum, Andreas Straube, Uwe Koedel, Louisa von Baumgarten

**Affiliations:** 1 Department of Neurology, University Hospital, LMU, Munich, Germany; 2 Institute for Clinical Neuroimmunology, University Hospital, LMU, Munich, Germany; 3 Department of Oncology, University Hospital, LMU, Munich, Germany; 4 Department of Neurology, HELIOS Amper-Hospital Dachau, Dachau, Germany

## Abstract

**Background:**

Early diagnosis of CNS lymphoma (CNSL) is essential for successful therapy of this rapidly progressing brain tumor. However, in patients presenting with focal brain lesions, fast and reliable diagnosis of PCNSL remains a challenge. A proliferation-inducing ligand (APRIL) and B cell activating factor (BAFF) are important factors in the pathophysiology, diagnosis, and prognosis of systemic B cell malignancies. However, their utility as biomarkers for the diagnosis of CNSL and their effects on CNSL cells remain unclear.

**Methods:**

In this prospective study, we analyzed the levels of APRIL and BAFF in the cerebrospinal fluid (CSF) of 116 patients with suspected focal brain lesions, including 53 CNSL patients. Additionally, we serially measured their levels during chemotherapy and relapse. Furthermore, we analyzed the effect of APRIL and BAFF on two B cell lymphoma cell lines using proliferation, viability, and chemotaxis assays.

**Results:**

CSF levels of APRIL and BAFF reliably differentiated CNSL from other focal brain lesions (including primary and metastatic brain tumors, autoimmune-inflammatory lesions, and neuroinfectious lesions) with a specificity of 93.7% (APRIL, BAFF) and a sensitivity of 62.3% (APRIL) and 47.1% (BAFF). Serial CSF analysis of CNSL patients during chemotherapy and relapse demonstrates a close correlation of APRIL CSF levels and the course of this disease. In vitro, APRIL and BAFF showed anti-apoptotic effects during MTX treatment and mediated chemotaxis of malignant B cells.

**Conclusion:**

This study extends the spectrum of valuable diagnostic biomarkers in CNSL. In patients with focal brain lesions, measurement of APRIL in CSF could help accelerating the diagnosis of CNSL. Moreover, our results highlight an important role of APRIL and BAFF in the pathophysiology of CNSL.

## Key points

- APRIL and BAFF levels are elevated in the CSF of CNS lymphoma patients, and APRIL alone or in combination with BAFF might serve as a diagnostic and therapeutic biomarker for these patients.
- APRIL and BAFF have anti-apoptotic effects and induce chemotaxis in B cell lymphoma cell lines.

## Introduction

Primary central nervous system lymphoma (PCNSL) is a rare type of extranodal non-Hodgkin lymphoma (NHL), accounting for 3% of all newly diagnosed primary intracranial tumors [1]. More than 95% of PCNSL are of the diffuse large B cell lymphoma subtype (DLBCL) [2]. Reliable diagnosis of this brain tumor remains a challenge. On MRI, PCNSL typically presents as contrast-enhancing cerebral lesions, often in direct contact with ependyma and/or meninges [1]. However, similar radiological features of PCNSL can be found in other intracranial tumors, as well as in lesions caused by demyelination, infection, vasculitis, or granulomatous disease [3]. For diagnosis of PCNSL, histological analysis of biopsy material, usually obtained by stereotactic biopsy, is regarded as gold standard. Up to 15% of PCNSL patients have leptomeningeal or ocular involvement, and lymphoma cells can be detected in the CSF or in the vitreous fluid using cytology, flow cytometry, or other molecular methods [4]. However, the latter does not allow a formal classification of PCNSL according to the new WHO criteria, which require immunohistochemistry for confirmation [5]. Nevertheless, several regions such as the brainstem and spinal cord cannot be safely accessed by stereotaxis [6]. Furthermore, up to 13% of patients require multiple stereotactic biopsies to establish diagnosis of PCNSL, in particular if steroid treatment has been given prior to biopsy. This can result in a therapeutic delay of up to several weeks, profoundly impeding the patients’ outcome [7]. Therefore, identification of reliable biomarkers in the CSF might help guiding the diagnosis of PCNSL. In HIV-positive patients with CNSL, Epstein-Barr virus DNA in the CSF can be used as a diagnostic biomarker [8]. In HIV-negative patients, several other biomarkers have been described, such as interleukin-10 [9–12], IL-6 [9, 10, 12], CXCL13 [11], neopterin [13], β-microglobulin [10, 14], osteopontin [15], sCD27 [16], specific microRNAs [17–21], and cell-free DNA [22], with varying diagnostic utility.

APRIL and BAFF are ligands of the tumor necrosis factor (TNF) family and play a key role in B cell survival, maturation, and differentiation. APRIL interacts with two receptors: transmembrane activator and CAML interactor (TACI) and B cell maturation antigen (BCMA), whereas BAFF interacts with both TACI, BCMA, and a third receptor, called BAFF-receptor (BAFF-R) [23, 24]. APRIL and BAFF have been linked to the pathophysiology of several autoimmune diseases, including systemic lupus erythematodes and multiple sclerosis [24–26]. Furthermore, recent clinical data indicate an important role for both ligands in systemic NHL: high serum levels of BAFF correlate with disease activity and poor response to treatment [27, 28]. Additionally, serum BAFF levels have been shown to represent an independent prognostic factor for overall and progression-free survival [27]. Similarly, increased APRIL expression in systemic B cell NHL correlates with lymphoma aggressiveness and inferior survival rate [29]. Recently, BAFF has also been implicated as a potential diagnostic biomarker in a small sample of PCNSL patients [30]. Also, two of its receptors, soluble TACI and soluble BCMA, are elevated in the CSF of PCNSL patients [31]. Furthermore, both BAFF and APRIL and their receptors are expressed in human PCNSL specimens [32]. However, the diagnostic utility of APRIL in PCNSL remains unclear.

In this study, we aimed to evaluate the diagnostic utility of APRIL and BAFF for CNS lymphoma. To this end, we performed a prospective analysis of APRIL and BAFF levels in the CSF of patients with focal brain lesions of different etiologies. Additionally, we analyzed the effect of APRIL and BAFF on proliferation, survival, and migration of two malignant B cell lymphoma cell lines in vitro and confirm their role in B cell lymphoma pathophysiology.

## Material and methods

### Study population

This monocentric, prospective study was conducted from February 2012 to June 2015 at the Department of Neurology, Klinikum Großhadern, Ludwig Maximilians University, Munich, Germany. The local ethics committee approved the study according to the Declaration of Helsinki (registration number 174-11). After written informed consent, patients (age ≥ 18 years) with at least one magnetic resonance imaging (MRI)-proven brain lesion of unknown origin (tumorous/autoimmune-inflammatory/infectious) were included into the study, if diagnostic lumbar puncture was performed during clinical routine. Our sample contained 30 PCNSL patients, of which 27 were diagnosed by stereotactic biopsy and subsequent histopathologic confirmation. In three patients with menineosis lymphomatosa, however, PCNSL was diagnosed using a combination of cytomorphology and flow cytometry. Most importantly, all patients received a whole body CT scan to rule out systemic lymphoma.

Also in 21 PCNSL patients with complete remission (CR) after methotrexate (MTX)-based polychemotherapy, CSF samples were analyzed. Furthermore, serial CSF analysis was done in 17 PCNSL patients at diagnosis as well as during and after MTX-based polychemotherapy. Additionally, 30 subsequent patients with other neurological diseases (ONDs) without a focal brain lesion were included as controls. After informed consent, samples of these patients have been collected prospectively during diagnostic work-up at the same department.

### CSF analysis

Immediately after collection, routine CSF analysis was performed at the Department of Laboratory Medicine, Ludwig Maximilian University, Munich, Germany. For enzyme-linked immunosorbent assay (ELISA) analysis, CSF was centrifuged immediately after collection and stored at − 80 °C.

### Cell lines

OCI-Ly10 cells were a kind gift from Prof. Dr. M. Dreyling. HKBML (human brain malignant lymphoma) cells [33] were obtained from RIKEN BioResource Center (Tsukuba, Ibaraki, Japan). OCI-Ly10 cells were cultured in Iscove’s modified Dulbecco’s medium (IMDM) (Life Technologies, Darmstadt, Germany) supplemented with 20% human plasma, 0.4% heparin, and 0.1% beta-mercaptoethanol. HKBML cells were cultured in Ham’s F12 Nutrient Mixture (Life Technologies, Darmstadt, Germany) supplemented with 15% fetal calf serum (FCS).

### ELISA

ELISA kits for APRIL were obtained from BioLegend (San Diego, USA). ELISA kits for BAFF were obtained from R&D Systems (Minneapolis, USA). Analyses were conducted according to the manufacturer’s instructions.

### Survival assays

Cells were cultured in serum-free medium (SFM) for 5 (OCI-Ly10) or 2 (HKBML) days, according to their sensitivity to serum starvation. Starved OCI-Ly10 (1 × 10^5^ cells/well) or HKBML cells (2 × 10^5^ cells/well) were cultured for 3 days in the presence of 500 ng/ml APRIL or BAFF protein (both R&D Systems, Minneapolis, USA) under SFM conditions at 37 °C in a humidified atmosphere (5% CO2 in air). Binding of APRIL and BAFF was blocked with 1 μg/ml TACI-Fc (R&D Systems, Minneapolis, USA) by pre-incubation for 30 min. OCI-Ly10 and HKBML cells (5 × 10^4^ cells/well) were treated with MTX (50 nM) in absence or presence of APRIL or BAFF. Blocking of pro-survival effects was performed by adding TACI-Fc (1 μg/ml). Cells were cultured for 3 days at 37 °C and 5% CO2 in a humidified atmosphere. All experiments were repeated at least 3 times.

### Chemotaxis assay

The in vitro migration of OCI-Ly10 and HKBM cells was evaluated using a 24-well Transwell assay (6.5 mm Transwell® with 5.0 μm Pore Polycarbonate Membrane Insert, Corning, Wiesbaden, Germany). APRIL and BAFF were diluted in SFM. Migration towards APRIL and BAFF was blocked with TACI-Fc (1 μg/ml) after pre-incubation for 30 min with 500 ng/ml of the respective protein. SFM served as control for baseline migration. Cells (1 × 10^6^ OCI-Ly10 cells/insert or 7 × 10^5^ HKBML cells/insert) were incubated for 5 h at 37 °C in a humidified atmosphere (5% CO2 in air). Migrated cells were counted on a Gallios Flow Cytometer (Beckman Coulter Inc., Brea, USA). All experiments were repeated at least 3 times.

### Statistics

Data are presented as median, median and range, or mean + SD, as indicated. The Shapiro-Wilk test was used to test for normal distribution, and in case of a non-normal distribution, the Kruskal-Wallis test was applied. The criteria for the post hoc test were adjusted using the Bonferroni correction. Outlier test was performed using Grubbs’ test. Spearman’s rank correlation was used for correlation analysis followed by Bonferroni’s correction to control for multiple testing. Wilcoxon’s test was applied for the comparison of paired samples, and the Mann-Whitney test for the comparison of unpaired samples. Sensitivity and specificity were analyzed using receiver-operating characteristic (ROC) analysis. Proliferation, survival, and chemotaxis results were compared by one-way analysis of variance followed by Tukey’s multicomparison test. A value of *P* < .05 was considered statistically significant. Statistical analysis were performed using the plugin “Real Statistics Using Excel” for Microsoft Office Excel (Zaiontz, C., 2014, version Rel 2.17.1.), and graphs were created using OriginPro 9.0 (OriginLab Cooperation, Northampton, MA, USA).

## Results

### Patient characteristics

One hundred sixteen patients with focal brain lesions were included into the study. Fifty-three of these patients were diagnosed with CNSL by histologic analysis. We also conducted serial CSF analysis in 17 CNSL patients before, during, and after MTX-based polychemotherapy. Furthermore, we acquired CSF samples from 21 CNSL patients in CR after MTX-based polychemotherapy. Detailed patient characteristics and the results of CSF analysis are summarized in Table 1.

**Table 1:** Characterization of the study population and the CSF parameters

Diagnosis	Patient characteristics			CSF parameters				
	*N*	Age	Sex (F/M)	Cells/μl	Atypical cells in *x*/*N*	Protein (mg/dl)	Glucose (mg/dl)	Albumin index CSF/serum [× 10^−3^]
Newly diagnosed PCNSL	30	70 (21–89)	13/17	7 (1–408)	8/30	67 (9–604)	64 (10–93)	10 (4–96)
Newly diagnosed SCNSL	7	66 (53–90)	2/5	9 (2–369)	1/7	73 (50–221)	54 (34–103)	13 (8–53)
Relapsed PCNSL	16	63 (23–84)	5/11	7 (1–202)	4/16	81 (38–448)	66 (12–99)	12 (3–65)
PCNSL complete remission	21	63 (27–78)	9/12	1 (0–8)	0/21	53 (21–106)	64 (51–88)	8 (2–49)
Primary brain tumor (PBT)	21	58 (21–83)	6/15	2 (0–204)	1/21	58 (25–160)	59 (53–89)	8 (2–22)
Metastatic brain tumor (MBT)	13	68 (31–77)	7/8	4 (0–80)	7/13	47 (15–191)	66 (45–98)	6 (4–13)
Autoimmune inflammatory disease (AID)	26	43 (19–72)	16/10	7 (0–144)	0/26	54 (24–116)	61 (51–101)	7 (2–19)
Neuroinfectious disease (NID)	3	47 (40–49)	1/2	7 (2–51)	0/3	66 (39–147)	56 (56–57)	13 (5–14)
Other neurologic disease (OND)	30	47 (18–85)	16/14	1 (0–3)	0/30	39 (22–83)	63 (47–91)	4 (3–12)

*Abbreviations*: *N* number, *F* female, *M* male. Data are presented as median (range). Detailed patient characteristics: 30 newly diagnosed PCNSL, all DLBCL; 27 diagnosed via histopathological confirmation, 3 diagnosed with flow cytometry and immunophenotyping. Twenty-seven immunocompetent, 3 human immunodeficiency virus (HIV) associated. Seven newly diagnosed SCNSL: 6 DLCBL, 1 marginal zone B cell lymphoma; all immunocompetent. Sixteen relapsed PCNSL: all DLBCL; all immunocompetent. Twenty-one primary brain tumors: 8 glioblastoma multiforme, 7 astrocytoma, 1 oligodendroglioma, 1 ependymoma, and 4 medulloblastoma. Thirteen metastatic brain tumors: 3 breast cancer, 3 lung cancer, 5 malignant melanoma, and 2 metastasis of unknown primary; 5 of these 13 patients were diagnosed with concomitant meningeosis neoplastica. Twenty-six autoimmune-inflammatory disease: 16 multiple sclerosis or clinically isolated syndrome at manifestation, 2 neuromyelitis optica, 3 acute disseminated encephalomyelitis, and 5 with stereotactic brain biopsy-proven autoimmune inflammation of unknown classification. Three neuroinfectious brain lesion: 1 fusobacterial brain abscess, 1 neurosyphilis, and 1 tuberculous meningoencephalomyelitis. Thirty other neurologic diseases without focal brain lesions: 17 headache, 4 neurodegenerative disease, 2 epilepsy, 2 myasthenia gravis, 2 normal pressure hydrocephalus, and 3 idiopathic facial palsy

### APRIL CSF levels in CNSL

**Fig. 1:**
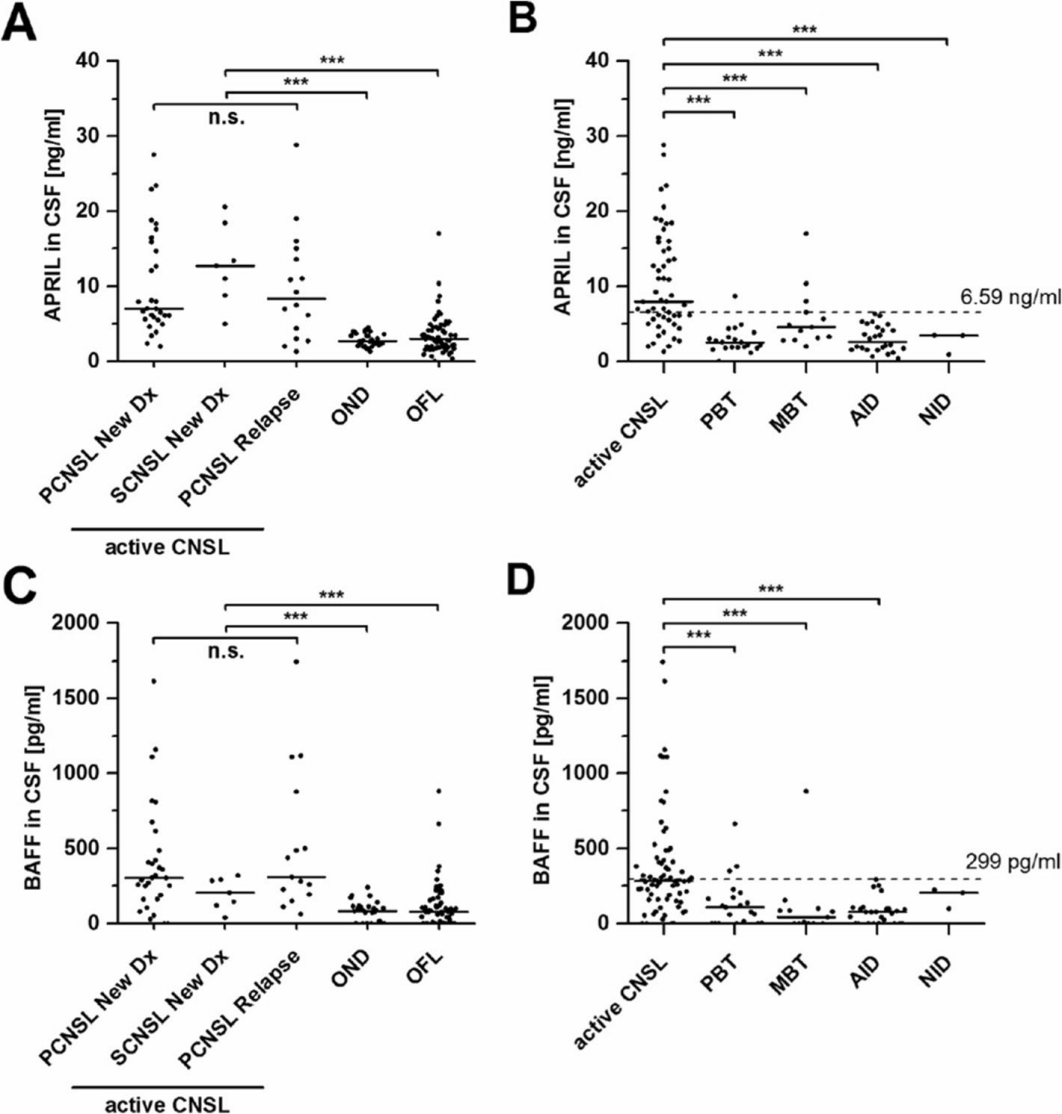
APRIL and BAFF are significantly elevated in CNSL. Median CSF levels of APRIL (**a**) and BAFF (**c**) do not significantly differ in patients with newly diagnosed (PCNSL New Dx) or relapsed PCNSL (PCNSL Relapse) and newly diagnosed secondary CNSL (SCNSL New Dx), taken together as *active* CNSL. All patients with active CNSL had significantly elevated APRIL CSF levels compared to patients with other neurological diseases (ONDs) and compared to patients with other focal lesions (OFLs). **b** Subgroup analysis of patients with OFL (PBT, primary brain tumor; MBT, metastatic brain tumor; AID, autoimmune-inflammatory disease; NID, neuroinfectious disease) demonstrates significantly elevated CSF levels of APRIL in patients with active CNSL compared to all other subgroups. **d** CSF levels of BAFF were significantly elevated in active PCNSL when compared to PBT, MBT, and AID, but not to NID. Black lines indicate median, and dotted lines represent the diagnostic cut-off value of 6.59 ng/ml (**b**, APRIL) and 299 pg/ml (**d**, BAFF) as calculated. n.s., not significant (*P* > .05). *P < .05; **P < .01; ***P < .001

APRIL CSF levels are illustrated in Fig. 1a, b. There were no significant differences in the CSF concentrations of APRIL in patients with primary, secondary, or relapsed CNSL (median 7.02 [range 2.00–27.52] vs. 12.71 [5.00–20.57] vs. 8.38 [1.32–28.82] ng/ml, *P* = .38) (Fig. 1a). For further analyses, these three patient groups were summarized as *active* CNSL. CSF APRIL levels of patients with active CNSL (8.0 [1.32–28.82]) were significantly higher compared to patients with ONDs (2.71 [1.31–4.50] ng/ml, *P* < .001) and to patients with any other focal lesion (OFL) (3.01 [0.00–17.01] ng/ml, *P* < .001) (Fig. 1a). Subgroup analysis revealed significant higher APRIL CSF levels in active CNSL (8.0 [1.32–28.82]) compared to patients with primary brain tumors (PBT, 2.55 [0–8.71] ng/ml, *P* < .001), metastatic brain tumors (MBT, 4.70 [2.00–17.01] ng/ml, *P* < .01), autoimmune-inflammatory diseases (AID, 2.61 [0.36–6.35] ng/ml, *P* < .001), and neuroinfectious diseases (NID, 3.44 [0.93–3.48] ng/ml, *P* < .01) (Fig. 1b).

**Fig. 2:**
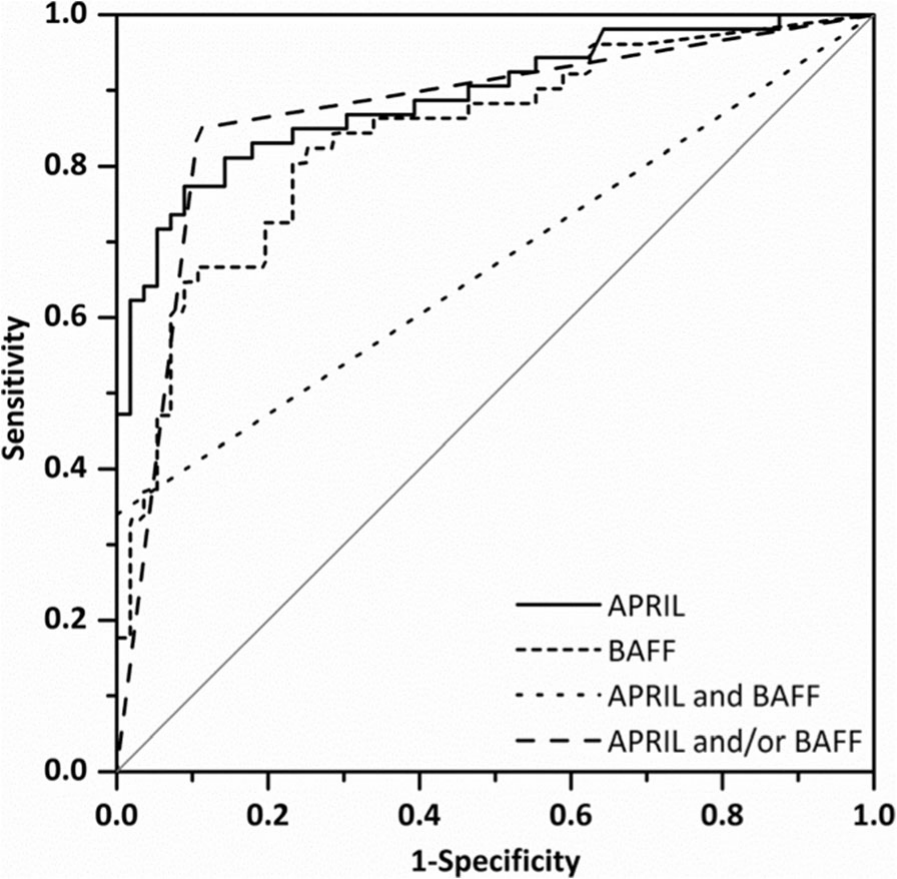
APRIL and BAFF CSF levels specifically discriminate CNSL from other brain lesions. Receiver operating characteristic (ROC) curve of 116 patients with focal brain lesions demonstrates a CSF level of APRIL > 6.59 ng/ml with specificity of 93.7% and a sensitivity of 62.3% for active CNSL (area under the curve [AUC], 0.866; 95% confidence interval [CI], 0.800–0.932). A CSF level of BAFF > 299 pg/ml exhibits a specificity of 93.7% and a sensitivity of 47.1% for active CNSL (AUC, 0.831; 95% CI, 0.755–0.910). Bivariate elevation of APRIL and BAFF in CSF had a specificity of 96.1% and a sensitivity of 30.2% for CNSL (AUC, 0.643; 95% CI, 0.543–0.743). Elevation of APRIL and/or BAFF in CSF showed a specificity of 96.1% and a sensitivity of 77.3% for diagnosis of active CNSL (AUC, 0.831; 95% CI, 0.753–0.910)

However, APRIL CSF levels elevated above the threshold of 6.59 ng/ml (as calculated by ROC analysis, Fig. 2) were found in three patients with metastatic brain tumors (two with lung adenocarcinoma (10.36 and 17.01 ng/ml) and one with malignant melanoma (8.04 ng/ml)). Notably, all of them had concomitant meningeal carcinomatosis. Furthermore, one patient diagnosed with glioblastoma multiforme showed an elevated APRIL CSF level (8.71 ng/ml). In this patient, a concomitant CSF inflammation with 240 lymphocytic cells/μl (but no malignant cells) was observed after stereotactic biopsy, suggesting a postoperative infectious reaction.

### BAFF CSF levels in CNSL

BAFF CSF levels are shown in Fig. 1c, d. No significant differences were detected between the BAFF CSF levels of patients with primary, secondary, or relapsed CNSL (311 [62–1742] vs. 205 [39–319] vs. 304 [0–1158] pg/ml, *P* = .59) (Fig. 1c). BAFF CSF levels in patients with active CNSL (292 [0–1742] pg/ml) were significantly higher compared to patients with ONDs (83 [0–243] pg/ml, *P* < .001) and to patients with OFLs (79 [0–880] pg/ml, *P* < .001). Subgroup analysis revealed significantly higher CSF BAFF levels in CNSL (292 [0–1742] pg/ml) compared to the subgroups of PBT (108 [0–661] pg/ml, *P* < .001), MBT (42 [0–880] pg/ml, *P* < .001), and AID (76 [0–293] pg/ml, *P* < .001) (Fig. 1d). In patients with neuroinfectious diseases, however, BAFF CSF level did not significantly differ from those of CNSL patients (205 [98–223] pg/ml, *P* = .16).

BAFF CSF levels were elevated above the threshold of 299 pg/ml (as calculated in ROC analysis, Fig. 2) in four patients with focal brain lesions other than CNSL: one patient with lung adenocarcinoma and concomitant meningeal carcinomatosis (880 pg/ml) and one patient with glioblastoma multiforme and concomitant CSF inflammatory syndrome (351 pg/ml); both of them also showed high APRIL CSF levels. In addition, two more patients in the primary brain tumor group had an elevation of BAFF: one with medulloblastoma (381 pg/ml) and one with astrocytoma and concomitant meningeal tumor spread (661 pg/ml).

### Correlation analysis

In all tested subgroups, correlation analysis was performed (Additional file 1: Figure S1A). In CNSL patients, no significant correlation was found between CSF levels of APRIL and BAFF. However, in patients suffering from PCNSL relapse, CSF APRIL levels correlated with CSF protein levels and albumin quotient, two markers for blood-brain barrier disruption (Qalb = CSF albumin/serum albumin). In patients suffering from primary brain tumors, CSF APRIL levels correlated to CSF cell number, CSF protein levels, and albumin quotient. Similarly, CSF BAFF levels of patients with metastatic brain tumors correlated to CSF cell number, CSF protein levels, and albumin quotient. Furthermore, in MBT patients, CSF APRIL levels were correlated to CSF BAFF levels. With age, no correlation was found in any of our tested subgroups. Of note, no association was found between CSF levels of APRIL or BAFF and the location of PCNSL (meningeosis, involvement of deep CNS structures, or direct contact to CSF as measured by MRI) (Additional file 1: Figure S1 B-G).

### Diagnostic potential of APRIL and BAFF

Given the elevation of APRIL and BAFF in the CSF of patients with active CNSL, we assessed the diagnostic utility of both proteins in the differentiation of CNSL from focal brain lesions of another origin using ROC analysis (Table 2). For the evaluation as a potential diagnostic biomarker with high clinical relevance, the often-used Youden’s index regards sensitivity and specificity as equally important. To specifically distinguish CNSL patients from other focal brain lesions in the clinical setting, however, specificity is a more useful parameter to rule out false positives [34]. ROC analysis revealed cut-off values for both proteins with a specificity of 93.7% (Fig. 2, Table 2). In all 116 patients with focal brain lesions, the elevation of APRIL CSF levels above the cut-off value of 6.59 ng/ml showed a sensitivity of 62.3% for the diagnosis of CNSL. For BAFF, a cut-off value of 299 pg/ml resulted in a sensitivity of 47.1%. An elevated level of CSF APRIL and/or BAFF was 96.1% specific and 77.3% sensitive; a bivariate elevation of both proteins was 96.1% specific and 30.2% sensitive.

**Table 2:** Sensitivity and specificity of two different CSF cut-off levels of APRIL and BAFF determined by ROC analysis

Cut-off CSF level	Sensitivity (%)	Specificity (%)
APRIL		
5.41 ng/ml (Youden’s Index)	77.4	87.3
6.59 ng/ml	62.3	93.7
BAFF		
121 pg/ml (Youden’s Index)	82.4	74.6
299 pg/ml	47.1	93.7

### APRIL and BAFF as therapeutic markers in PCNSL

**Fig. 3:**
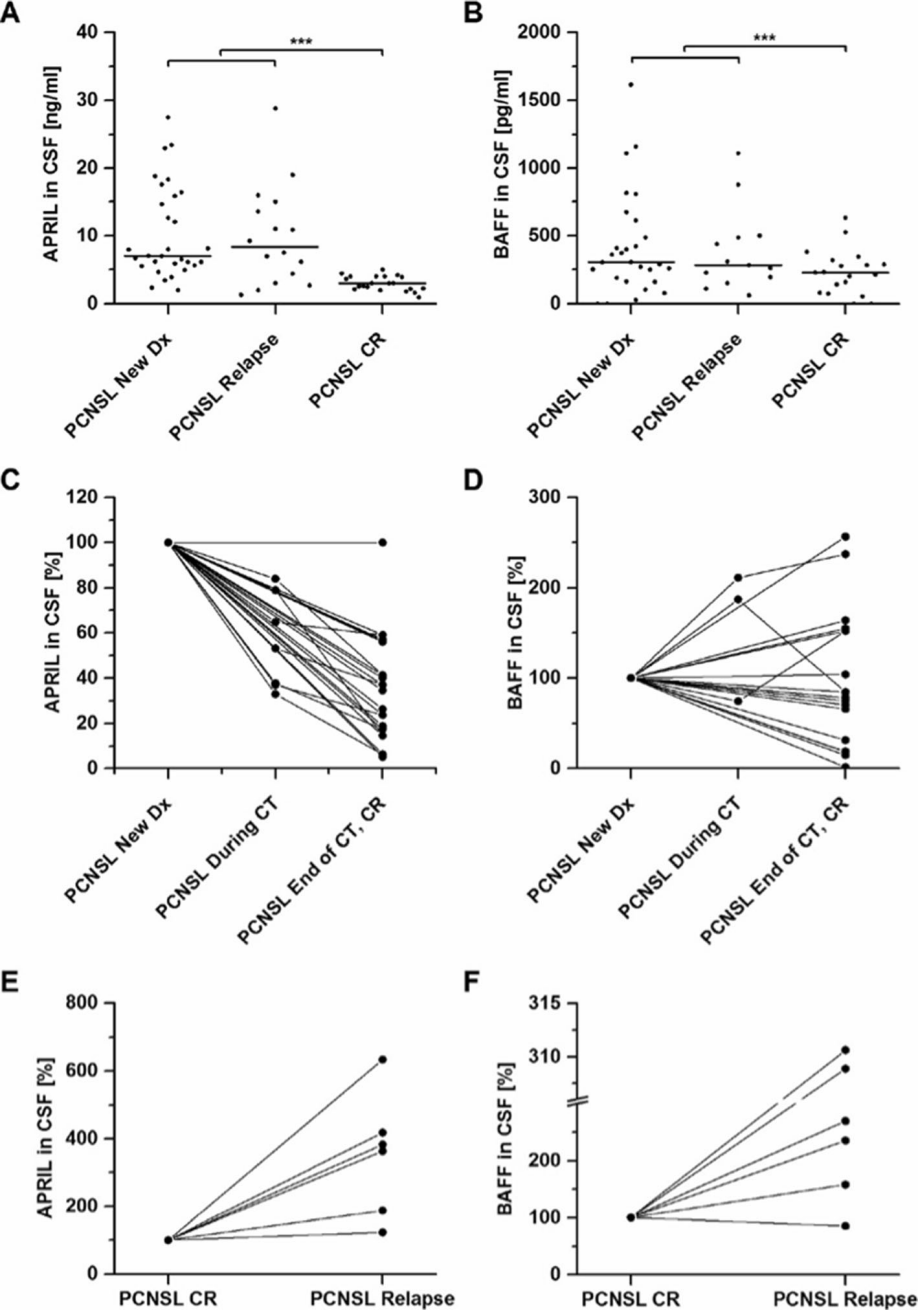
APRIL and BAFF CSF levels in PCNSL during treatment. Patients with complete remission after methotrexate (MTX)-based polychemotherapy (*n* = 21) had significantly lower APRIL (**a**) and BAFF (**b**) CSF levels compared to patients with untreated newly diagnosed or relapsed PCNSL (*n* = 46). **c**–**f** Serial CSF analysis for APRIL (**c**, **e**) and BAFF (**d**, **f**) was performed before, during, and after methotrexate (MTX)-based polychemotherapy in 17 patients with newly diagnosed PCNSL (new DX). All of them experienced complete remission (CR) at the end of chemotherapy (CT). In 6 of these patients, relapse occurred during the observation period. **c** APRIL CSF levels decrease in 16/17 patients (94%) at CR, while in 1/17 patients (6%), APRIL levels remained stable (mean decrease: 63%). **d** BAFF CSF levels show variable courses during treatment and at CR (decrease in 10/17 patients (59%), increase in 6/17 (35%), stable in 1/17 (6%) with a mean decrease of 4%). **e** At relapse, APRIL CSF levels increase in 6/6 patients to a mean of 351% (range 123 to 633%. **d** BAFF CSF levels increase in 5/6 cases to a mean of 228% (range 85 to 311%)

To evaluate the role of APRIL and BAFF as therapeutic biomarkers in PCNSL, we examined their CSF levels in 46 patients with newly diagnosed (untreated) or relapsed PCNSL and in 21 patients with CR after MTX-based polychemotherapy. Compared to patients with untreated disease, patients with CR showed significantly lower levels of both APRIL and BAFF CSF levels (APRIL 2.80 [0.95–5.00] vs. 7.28 [1.32–28.82] ng/ml, *P* < .001, and BAFF (230 [0–633] vs. 307 [0–1742] pg/ml, *P* < .001) (Fig. 3a, b). The reduction of BAFF CSF level, however, was less pronounced and showed a higher variability. Of note, no association was found between the location of PCNSL (meningeosis or involvement of deep CNS structures) and CSF levels of APRIL or BAFF (Additional file 1: Figure S1).

**Fig. 4:**
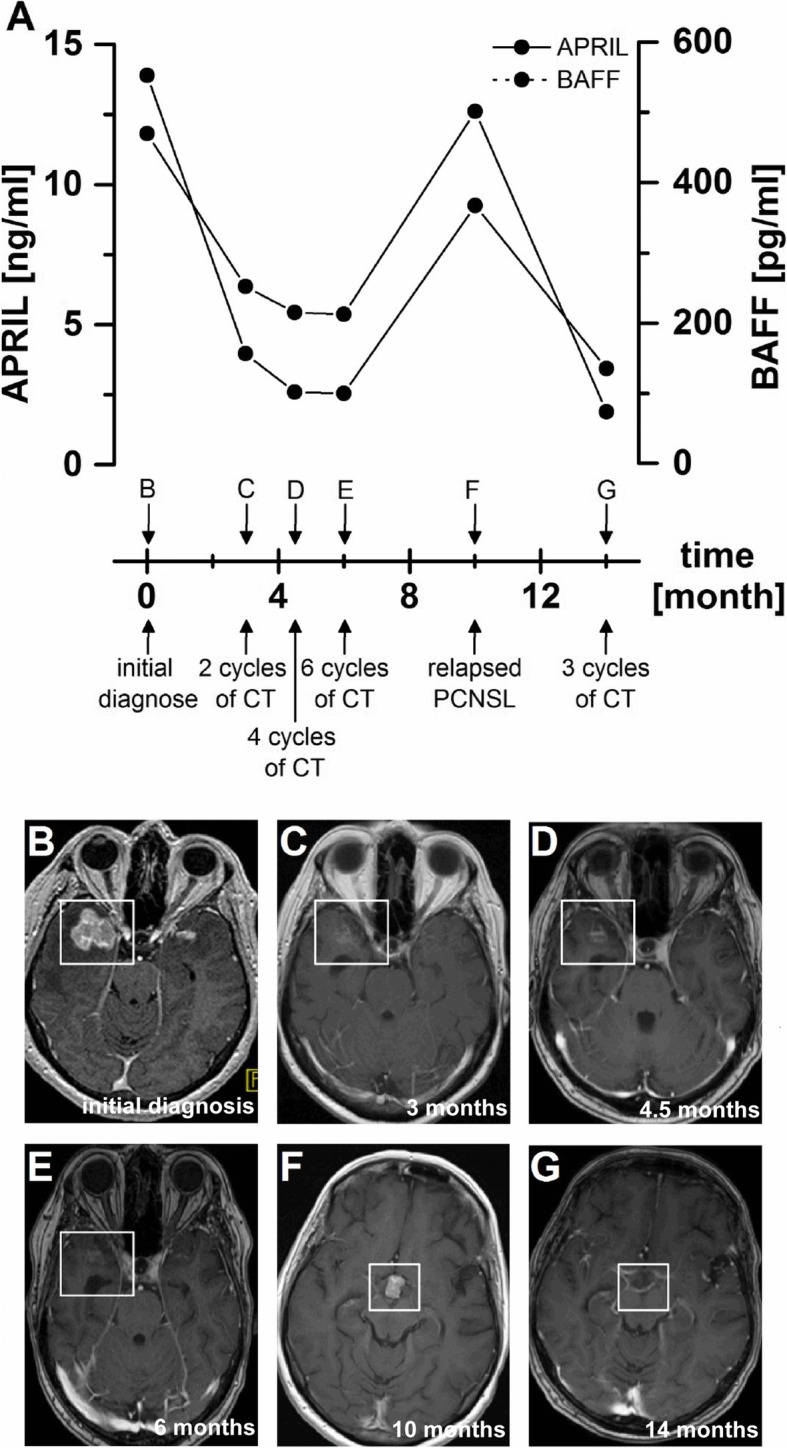
APRIL and BAFF CSF levels are associated with treatment response and relapse—a representative patient example. CSF levels of APRIL and BAFF (**a**) and corresponding, representative MRI images (**b**–**g**, T1 after gadolinium) of a 61-year-old female patient with newly diagnosed multilocular PCNSL in the right temporal lobe, the left posterior splenium, the left temporopolar lobe, and meningeosis lymphomatosa with basal meningeal enhancement over a period of 14 months. Initially, CSF levels of APRIL and BAFF decrease during 6 cycles of MTX-based polychemotherapy (CT), corresponding to radiological response (**b**–**e**). At relapse, APRIL and BAFF CSF level increase (**a**), while MRI (**f**) shows relapsed disease next to the third ventricle, which subsided after 3 (of 6) cycles of repeated CT (**g**). Correspondingly, APRIL and BAFF CSF levels decreased (**a**)

Next, we determined APRIL and BAFF CSF levels in 17 patients with newly diagnosed or relapsed PCNSL before, during, and after MTX-based polychemotherapy. Here, APRIL proved to be a marker of response to treatment: 16/17 patients (94%) showed decreased CSF levels of APRIL during CR with an average reduction of 63% (Fig. 3c). In 6 PCNSL patients after CR, we assessed CSF levels at relapse and observed an increase of APRIL CSF levels in 6/6 patients with a mean increase of 351% (Fig. 3e). In contrast, serial analysis of BAFF CSF levels in the same 17 patients before, during, and after chemotherapy did not conclusively correlate with the disease course: in some patients a treatment-related decrease could be observed at CR (*n* = 10), whereas other patients revealed stable or even increased BAFF CSF levels (*n* = 7) (Fig. 3e). At disease relapse, BAFF CSF levels rose in 5 of 6 cases to a mean increase of 228% (Fig. 3f). Figure 4 shows a representative serial analysis of a 61-year-old female patient with newly diagnosed PCNSL. In this case, APRIL and BAFF CSF levels correlated well with clinical and radiological responses to treatment and disease relapses.

### Pathophysiologic relevance of APRIL and BAFF in PCNSL

**Fig. 5:**
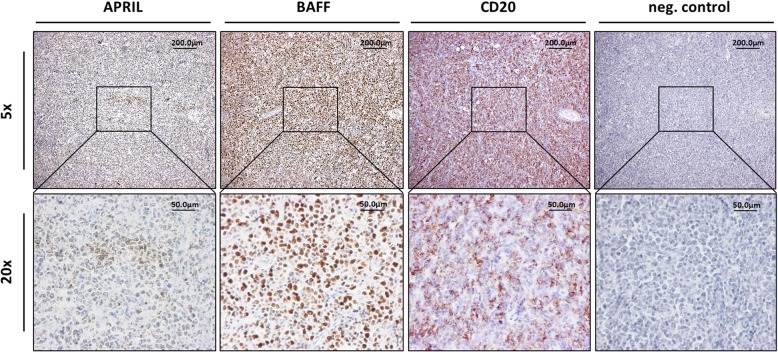
Representative immunohistochemistry demonstrating high expression of BAFF and variable expression of APRIL in a patient with newly diagnosed PCNSL. Negative control was performed from the same tissue by omission of primary antibodies. Scale bar, 200 μm (upper panels) and 50 μm (lower panels). Other samples of this patient have already been published before [32]

As Birnbaum et al. have already shown before, BAFF is highly expressed in PCNSL, while APRIL shows variable expression [32]. A representative example of a patient with newly diagnosed PCNSL (DLBCL subtype) is shown in Fig. 5.

**Fig. 6:**
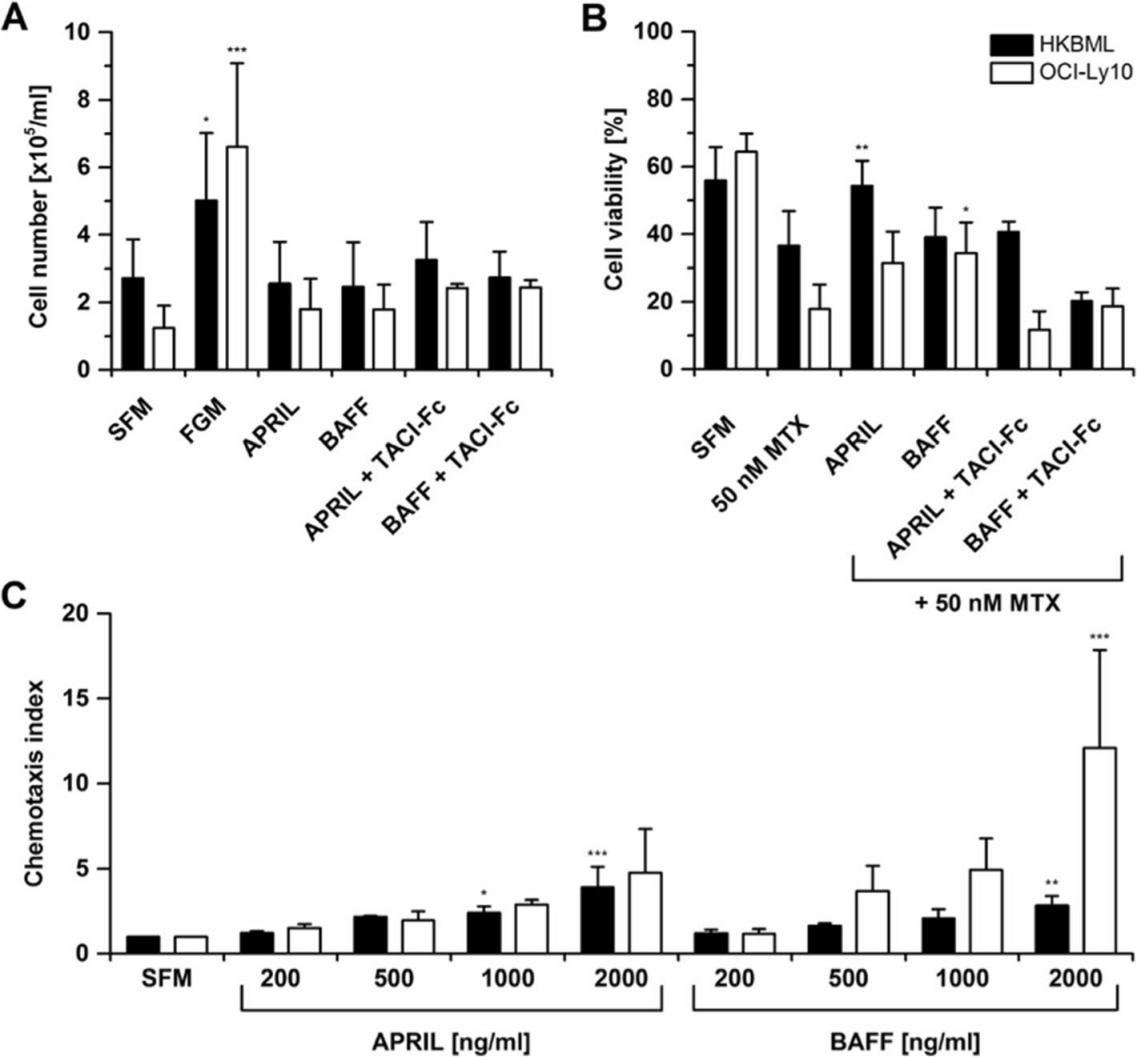
APRIL and BAFF increase cell survival during MTX treatment and induce chemotaxis in lymphoma cells in vitro. **a** In full growth medium (FGM), cell proliferation is increased compared to serum-free medium (SFM). APRIL and BAFF did not affect the proliferation of HKBML or OCI-Ly10 cells when added to SFM. **b** Under MTX exposition in SFM, the addition of APRIL significantly increased the tumor cell viability of HKBML cells (*P* < .01), while the addition of BAFF significantly increased the tumor cell viability of OCI-Ly10 cells (*P* < .05). The APRIL- and BAFF-neutralizing TACI-Fc abrogate these APRIL- and BAFF-mediated effects. **c** OCI-Ly10 and HKBML cells exhibit increased chemotaxis to BAFF, and HKBML cells also to APRIL in a dose-dependent manner. Chemotaxis index is defined as migrated cells after addition of chemokine normalized to the number of spontaneously migrated cells in SFM. *P < .05; **P < .01; ***P < .001

To analyze their effects on lymphoma cells in vitro, we used the systemic DLBCL cell line OCI-Ly10 and the human primary CNSL cell line HKBML [33]. The presence of TACI, BCMA, and BAFF-R was confirmed by immunohistochemistry (data not shown), indicating that these cells are fully equipped to respond to APRIL and BAFF. In vitro, APRIL and BAFF showed no effects on proliferation (Fig. 6a) or viability (Fig. 6b) in serum-free medium. However, APRIL significantly increased cell survival under MTX exposition in HKBML cells, whereas BAFF promoted cell survival under MTX exposition in OCI-Ly10 cells (Fig. 6b), indicating anti-apoptotic effects. The inhibitor TACI-Fc blocked the pro-survival effects mediated by APRIL and BAFF (Fig. 6b). Furthermore, a dose-dependent chemotactic effect of both, APRIL and BAFF, on the migration of OCI-Ly10 and HKBML cells was observed (Fig. 6c).

## Discussion

In this prospective study, we illustrate the potential of APRIL and BAFF as reliable diagnostic and therapeutic biomarkers in CNSL in a large patient cohort. Furthermore, we demonstrate how both factors might contribute to CNSL pathobiology.

In our prospectively collected sample, the 53 patients with primary and secondary CNSL showed significantly elevated APRIL and BAFF CSF levels compared to patients with other neurological diseases. Most importantly, it reliably helped to discriminate CNSL from other focal brain lesions, mimicking the radiographic appearance of CNSL. With considerable sensitivity and specificity, APRIL alone, or in combination with BAFF, seems to be well suited to serve as a simple and reliable diagnostic biomarker for CNSL.

### Other biomarkers

Several other potential biomarkers for PCNSL have been identified in the past. The proteins neopterin [13], CXCL13 [11], IL-10 [9–12], IL-6 [9, 10, 12], β-microglobulin [10, 14], osteopontin [15], BAFF [30], sTACI and sBCMA [31], and sCD27 [16] have been analyzed for their potential as diagnostic biomarkers. Their reported sensitivities for the diagnosis of PCNSL ranged from 68% (ß2-microglobulin [14]) to 100% (sCD27 [16]) with specificities ranging from 63% (IL-6 [9]) to 100% (IL-10 [9]). However, direct comparison across studies is often challenging, as important factors often vary widely. These factors include the study design, the composition of the control cohort (control patients with focal brain lesions of malignant, inflammatory, and infectious origin; control patients without focal lesions; healthy controls), the quantification method, the chosen cut-off level, and the number of patients included. For example, Mizutani et al. recently present the value of BAFF and TACI as a biomarker for CNSL with a reported sensitivity and a specificity of 100% [30]. However, their retrospectively collected data included only nine PCNSL patients. Additionally, also, the reported diagnostic potential of several biomarkers (e.g., IL-10 [10, 11]) varied when analyzed by different groups. A recent meta-analysis concludes that due to these methodological issues (heterogeneous sample selection and test definitions, mostly retrospective small-scale studies), currently, none of the reported biomarkers shows a sufficient level of evidence to replace the gold standard of brain biopsy [34]. However, more prospective studies, together with validation of these markers in consecutive cohorts, were recommended, ideally measuring several or all of the identified markers simultaneously. Thereby, biomarker profiles of patients, based on several different biomarkers, could be created with potentially higher diagnostic accuracy than single markers.

In addition to protein biomarkers, several microRNAs, quantified by quantitative real-time PCR, have been analyzed as biomarkers in the diagnosis of different brain tumors, including PCNSL [17–21]. In this context, microRNAs miR-21, miR-19b, and mirR-92a [17, 35] and U2 small nuclear RNA [36] enabled the differentiation of patients with PCNSL from controls with considerable sensitivity and specificity. Furthermore, microRNA profiles or selected microRNAs in the peripheral blood of PCNSL patients seem to be associated with survival of patients under therapy [37–39]. In addition, the detection of tumor-specific cell-free DNA in the CSF of CNSL patients shows promise as a reliable marker in a subset of CNSL patients [22]. Similarly, other studies have been published about cell-free tumor DNA in the blood or in vitreous biopsies of CNSL patients [40–42], illustrating the feasibility to detect tumor-specific mutations in these patients. These findings appear very promising and might pave the way for potential targeted therapies against CNSL based on their molecular signature (e.g., Bruton’s tyrosine kinase (BTK) inhibitors for MYD88-mutated tumors [43]). However, their widespread use as diagnostic biomarkers is still severely limited by their limited sensitivity, ranging from 24 to 59%. Additionally, CNSL-specific mutations are not known, as mutations present in CNSL patients can also be present in other diseases such as Waldenström macroglobulinemia, IgM-MGUS, or marginal zone lymphoma [44]. Therefore, these studies warrant further validation in prospective trials including bigger patient cohorts before its use as a diagnostic biomarker can be recommended.

In the context of the abovementioned CSF biomarker studies, our prospective study is comprised of a large, clinically relevant patient cohort and includes a broad spectrum of control patients with neoplastic, inflammatory, or infectious brain lesions, representing a relevant differential diagnosis in patients with PCNSL.

### Utility of APRIL and BAFF CSF levels as diagnostic and therapeutic biomarkers for CNSL

We could include 116 patients with focal brain lesions. Fifty-three of those were histologically diagnosed with CNSL. An elevated CSF level of APRIL is a promising diagnostic marker for CNSL (specificity 93.7% and sensitivity 62.3%). The combination of both markers (elevation of BAFF and/or APRIL) further increases the diagnostic accuracy (specificity 96.1%, sensitivity 77.3%). APRIL and BAFF CSF detection could therefore be a reliable, simple, and cost-efficient way to establish the diagnosis of CNSL in patients with focal brain lesions that cannot safely be assessed by stereotactic biopsy or in cases in which biopsy failed to establish the diagnosis. However, our results have to be validated in an independent cohort. Special consideration should be given to the differential diagnosis of herpes simplex virus (HSV) encephalitis. In our prospective study population with focal brain lesions, no patient suffering from HSV encephalitis was included. However, these patients can present with focal contrast-enhancing brain lesions, and increased CSF levels of APRIL and BAFF above our presented cut-off values have been described (median levels of 19 ng/ml and 590 pg/ml, respectively) [45]. Therefore, this potentially fatal disease must always be considered as an important differential diagnosis.

Furthermore, we illustrate the utility of APRIL as a therapeutic biomarker, as CSF levels correlate with response to MTX-based polychemotherapy and disease relapse in our sample. However, validation studies are needed before definite conclusions can be drawn regarding APRIL as a therapeutic biomarker in clinical practice. BAFF failed to be a reliable marker of response to treatment or recurrent disease. In systemic NHL and rheumatic diseases, BAFF serum levels increase during treatment with rituximab [46–48]. Treatment regimens for CNSL containing rituximab might also affect BAFF CSF levels and could explain the inconsistent CSF BAFF levels during the disease course in our CNSL patients. Although we do not present long-term data, it is tempting to speculate that APRIL and/or BAFF CSF levels could also serve as prognostic indicators. However, further studies are needed to evaluate this hypothesis.

Detectable levels of APRIL and BAFF were also found in patients with other focal brain lesions. In patients with multiple sclerosis, the detection of APRIL and BAFF in CSF is well established [49–51] and within the range of our data. In this disease, both ligands regulate inflammatory processes within the demyelinating lesions [52]. However, APRIL and BAFF CSF levels in patients with autoimmune-inflammatory diseases were significantly lower than in patients with CNSL.

In our study, we found elevated APRIL and/or BAFF levels above the cut-off value in 7 of 63 patients with malignant brain tumors. Four of these patients showed leptomeningeal tumor spread (two patients with lung carcinoma, one patient with malignant melanoma, and one patient with astrocytoma), and one patient showed signs of postoperative infection after stereotactic biopsy (glioblastoma multiforme).

### Pathophysiological relevance of APRIL and BAFF

In a previous study, we could confirm the expression of APRIL and BAFF in PCNSL lesions [32]. However, their exact cellular origin in CNSL remains unclear. It might be released by lymphoma cells themselves and/or be produced by its reactive, inflammatory microenvironment. Monocytes, macrophages, and T cells as well as microglia and reactive astrocytes are all able to secrete APRIL and/or BAFF [26, 53, 54].

The expression of APRIL and BAFF is also relevant for other tumors than CNSL. In several types of systemic carcinomas, the overexpression of APRIL and BAFF was associated with tumor progression and tumor grade. APRIL overexpression was found in invasive bladder carcinoma, adult germ cell tumor, and adenocarcinoma of the esophagus and pancreas. BAFF was found overexpressed in breast carcinoma, adenocarcinoma of the esophagus, and adult germ cell tumor [55]. Also in gliomas, the expression of APRIL and BAFF and their cognate receptors (BCMA, TACI) correlated with tumor grade [56]. Furthermore, APRIL was shown to promote the proliferation of human glioblastoma cell lines [57].

In systemic lymphomas, both TNF superfamily members are regulators of the balance between cell survival and programmed cell death. There, its overexpression results in the increased survival of malignant B cells [58–60] via activation of the NF-κB pathway [61–63]. APRIL [63] and BAFF [59, 63] have been shown to promote survival of primary tumor cells, isolated from patients with untreated B-CLL, in an autocrine pathway. Also in vitro, malignant B cells isolated from patients with NHL as well as different NHL cell lines have been shown to be more resistant to apoptosis after the addition of APRIL and BAFF [64, 65].

Further studies support the possible pathophysiological effect of APRIL and BAFF on PCNSL, as not only APRIL and BAFF, but also their receptors BAFF-R, BCMA, and TACI are expressed in CD20-positive tumor cells of human PCNSL specimen [26, 32]. Additionally, the reported increase in CSF levels of sBCMA and sTACI of PCNSL patients further supports this hypothesis [31], as these soluble receptor levels at least partly reflect the level on the cell surface [66, 67].

In this study, we show that APRIL reduced MTX-induced apoptosis in a B cell CNS lymphoma cell line, while BAFF reduced MTX-induced apoptosis in a systemic B cell lymphoma cell line, indicating pro-survival effects for both ligands in B cell lymphoma. Interestingly, the addition of TACI-Fc abrogated both of these pro-survival effects. These findings corroborate the importance of APRIL and BAFF signaling for MTX-based chemotherapy against this disease. Further, we provide evidence that APRIL and BAFF induce chemotaxis of systemic and CNS lymphoma cell lines, confirming previously published results showing that BAFF increases the chemotactic response of human B cells to CXCL12, CXCL13, and CCL21 in vitro [68].

So far, it remains unclear whether CNSL arises in the brain or whether malignant B cells escape the systemic immunosurveillance and proliferate in this immunoprivileged site. In both cases, it seems possible that APRIL and BAFF may contribute not only to the survival but also to the CNS tropism of malignant B cells. Furthermore, anti-BAFF therapies are already being used clinically in the therapy of SLE [69], and it is tempting to speculate that they could also be of potential use in the therapy of PCNSL.

## Conclusion

CSF levels of APRIL could help establishing the diagnosis of CNSL, especially if stereotactic biopsy is impossible or not conclusive. Moreover, APRIL can serve as a therapeutic marker during therapy and relapse. Future studies with larger patient cohorts are needed to confirm our results and to analyze whether these proteins might have also prognostic value. Furthermore, APRIL and BAFF have pro-survival effects on CNSL cells during chemotherapy in vitro and, if confirmed in additional studies, might represent a novel therapeutic target for the treatment of CNSL.

## Supplementary information

**Additional file 1: Figure S1.** Correlation of CSF APRIL and BAFF levels with CSF and MRI features. (A) Correlations of CSF levels of APRIL and BAFF with CSF cell count, CSF glucose, CSF protein, CSF albumin quotient and age were calculated (Spearman correlation; an adjusted *p*-value of *p* = 0.0083 was calculated by Bonferroni correction to control for multiple testing). (B-G) CSF levels of APRIL and BAFF show no difference when grouped according to presence of meningeosis (B, C), involvement of deep structures (D, E), or direct contact to CSF (F, G). Mann-Whitney U test, n.s., not significant.

## Data Availability

The datasets analyzed during the current study will be made available from the corresponding author on reasonable request.
