# Supplementary figures and images for: APRIL and BAFF: novel biomarkers for central nervous system lymphoma
Source: J Hematol Oncol. 2019 Oct 15;12:102. doi: 10.1186/s13045-019-0796-4 (PMC6792247; doi:10.1186/s13045-019-0796-4)

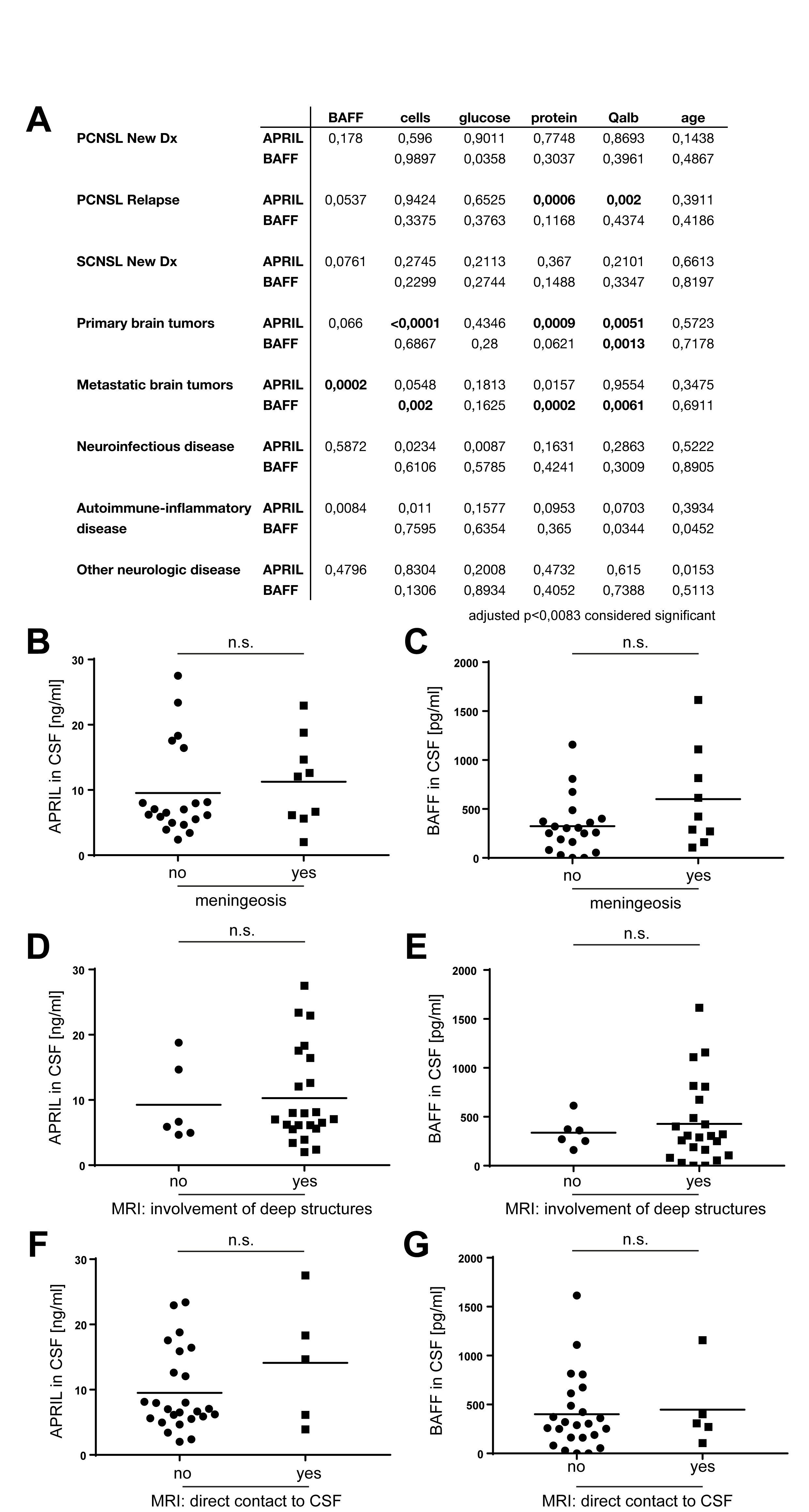

Supplement: Supplementary file 1 — Additional file 1: Figure S1. Correlation of CSF APRIL and BAFF levels with CSF and MRI features. (A) Correlations of CSF levels of APRIL and BAFF with CSF cell count, CSF glucose, CSF protein, CSF albumin quotient and age were calculated (Spearman correlation; an adjusted p-value of p = 0.0083 was calculated by Bonferroni correction to control for multiple testing). (B-G) CSF levels of APRIL and BAFF show no difference when grouped according to presence of meningeosis (B, C), involvement of deep structures (D, E), or direct contact to CSF (F, G). Mann-Whitney U test, n.s., not significant. [file 13045_2019_796_MOESM1_ESM.tif]
